# Editorial

**Published:** 1838-05-23

**Authors:** 


### THE MEDICAL EXAMINER.

PHILADELPHIA, MAY 23, 1836.

We promised our readers, some time since, a notice of Dr. Ryan's Philosophy of Marriage. This work is a production of a different character, from what we had hoped, and supposed we had a right to expect to find it. It is nominally addressed to the medical profession, but is, in reality, intended to pander to the tastes of that portion of the public, to whom the supposed mysteries, which Dr. Ryan unveils, are matters of curiosity. The subjects of the book are professedly the philosophy of marriage, in its social, moral, and physical relations, with an account of the diseases of the genito-urinary organs, which impair or destroy the reproductive function, and induce a variety of complaints; with the physiology cf generation in the vegetable and animal kingdoms. Some other matters, perhaps the most *piquant* portion of the work, are included under the head of a part of a course of obstetric lectures, delivered by the author. All these topics are treated with a freedom which has never been before equalled in a professional writer, and which could, we think, be with difficulty surpassed. We have the whole subject of marriage discussed, from its moral and social features, down to minute details upon the "chaste mysteries of hymen," which the novelist forebore to, but the physician does not scruple to profane. We shall not, of course, enter into any of the interesting points which Dr. Ryan treats of, nor serve up scraps of his *haut-goût* anecdotes. We have noticed his work, only to condemn it: it is utterly unworthy of analysis or criticism, and deserves only unqualified censure.

Licentious writings from a physician are an inexcusable outrage upon society, which can most justly turn upon him, with the *et tu!* Another offence, of this character, of an inferior grade, indeed, but sufficiently heinous, and more common, is the freedom of language and topic, in which certain teachers of medicine indulge in their lectures to to their pupils. An ample latitude for the discussion of scientific subjects is allowable, but there can be no justification for grossness of expression, or the delivery of obscene and generally irrevelant anecdotes. There is evil enough committed, if there be no other, when an assemblage of youth from the different quarters of our country, are led to presume, as they must, the metropolitan tone of manners to be tolerant of the indelicacy of speech, which an individual of age and station is seen habitually to resort to. It is a mistake, too, to suppose that popularity is obtained by this line of conduct. They, who thus court it, should learn to distinguish between the smile of applause and the smile of contempt. We would pray them, then, for their own sakes, to amend a behaviour, which, though it may make the unskilful laugh, cannot but make the judicious grieve—if there be, indeed, any so unskilful as to laugh at so sorry an exhibition.

